# An Electroencephalography Network and Connectivity Analysis for Deception in Instructed Lying Tasks

**DOI:** 10.1371/journal.pone.0116522

**Published:** 2015-02-13

**Authors:** Yue Wang, Wu Chun Ng, Khoon Siong Ng, Ke Yu, Tiecheng Wu, Xiaoping Li

**Affiliations:** Neuroengineering Lab, Mechanical Engineering Department, National University of Singapore, Singapore, Singapore; University of British Columbia, CANADA

## Abstract

Deception is an impactful social event that has been the focus of an abundance of researches over recent decades. In this paper, an electroencephalography (EEG) study is presented regarding the cognitive processes of an instructed liar/truth-teller during the time window of stimulus (question) delivery period (SDP) prior to their deceptive/truthful responses towards questions related to authentic (WE: with prior experience) and fictional experience (NE: no prior experience). To investigate deception in non-experienced events, the subjects were given stimuli in a mock interview scenario that induced them to fabricate lies. To analyze the data, frequency domain network and connectivity analysis was performed in the source space in order to provide a more systematic level understanding of deception during SDP. This study reveals several groups of neuronal generators underlying both the instructed lying (IL) and the instructed truth-telling (IT) conditions for both tasks during the SDP. Despite the similarities existed in these group components, significant differences were found in the intra- and inter-group connectivity between the IL and IT conditions in either task. Additionally, the response time was found to be positively correlated with the clustering coefficient of the inferior frontal gyrus (44R) in the WE-IL condition and positively correlated with the clustering coefficient of the precuneus (7L) and the angular gyrus (39R) in the WE-IT condition. However, the response time was found to be marginally negatively correlated with the clustering coefficient of the secondary auditory cortex (42L) in the NE-IL condition and negatively correlated with the clustering coefficient of the somatosensory association cortex (5L, R) in the NE-IT condition. Therefore, these results provide complementary and intuitive evidence for the differences between the IL and IT conditions in SDP for two types of deception tasks, thus elucidating the electrophysiological mechanisms underlying SDP of deception from regional, inter-regional, network, and inter-network scale analyses.

## Introduction

Deception, a complex human behavioral manifestation, is impactful when causing serious safety threats and economic harm to the society. To counteract these potential threats, lie detection has been used in criminal investigations, employee honesty pre-screenings and forensic settings. Unfortunately, existing methods lack sensitivity and specificity mainly due to the fact that the underlying deception mechanism is poorly understood. In contrast to the polygraph [1] that measures deception’s peripheral manifestation, EEG and fMRI directly measure the organ that generates the deceptive activities. Most previous studies have been investigating whether there are generic neuronal finger-prints for liars. The previous fMRI studies have shown important roles of prefrontal and anterior cingulated cortex (ACC) in deception [2–5], highlighting ‘executive control’ as a core component of deception. Apart from this, the modulation effect of deception on the EEG power spectrum has been observed on the scalp level in previous studies [6, 7], specific EEG frequency band (i.e. alpha) was associated with lying, demonstrating that risk monitoring/expectation and increased cognitive load play important roles in deception. In addition, some previous EEG studies focused on a frequently used ERP-based paradigm [8], i.e. to detect concealed information in the ‘guilty knowledge test’(GKT) [9] that depends on the detection of a well-known P300 ‘oddball’ response [10] which basically detect liars’ response to salient targets. Although quite a few regions of interest/frequency band/ERP peak have been identified, the complete underlying of deception mechanism remains obscure. Unfortunately, no attempt has been taken before to unveil the functional networks underlying deception related processes, given the fact that deception is such a complicated cognitive process. Despite the challenge, in theory, neuronal communication should play an important role in deception related processes which involves coordination between different brain areas that belong to particular functional networks. Hence, it is interesting to take an attempt to investigate the network activities in the same frequency band from which distinct event related synchronization (ERS) was found (i.e., upper alpha band) in our previous study [11].

In contrast to previous studies that focused on the time window during which deceptive response is being generated, this study investigated the window prior to that period (i.e. stimulus delivery period (SDP)). The underlying hypothesis is that before the subjects giving any deceptive/truthful responses, their corresponding neuronal activities may already start to differentiate from each other, which possibly indicates the existence of a prediction sign of deception. Understanding the networks underlying deception during this time window possibly enables us to convert this whole complicated process into more easily understood cognitive processes, and to explore the potential earlier deception sign that has never been discussed before.

In this study, commonalities and differences between two types of instructed lying tasks during SDP were investigated. Interestingly, compared with the ‘with prior-experience’ lying task (WE), the ‘non-experience’ lying task (NE) remains largely uninvestigated. Previous deception studies have focused on comparisons between deception types such as instructed/spontaneous lying [7], self-referential/other-referential lying [12], and memorized/created lying [2]. Limited work has been carried out on comparisons between deception in experienced and non-experienced events. Among the fMRI studies, one study [3] instructed subjects to tell lies/the truth concerning 20 old stimuli and 20 new stimuli by answering ‘I know’ and ‘I don’t know’. This fMRI study considered both experienced and non-experienced events in the deception tasks without requiring fabrication of lies to the unfamiliar objects and the neural circuits underlying the processing were dominated by recognition memory and inhibition control. However, real-life deception for non-experienced events often involves fabricating lies while being asked questions that require semantic processing especially in the interview scenario. In general, this fMRI study remains distinct from the NE task that induces subjects to cheat with extra efforts in semantic processing. The motivation to study these two tasks is based on the further hypothesis that, features that can distinguish the IL and IT during the SDP may be different in varying tasks and when features of lying during the SDP is being discussed, it could be task-dependent.

In summary, this study is aimed to develop a new EEG-based approach to explore the potential neural mechanism of instructed lying during SDP that could potentially provide a basis for future applications. Additionally, previous EEG-based studies did not provide a neuro-anatomy basis for deception, and previous protocol designs for instructed lying may have only considered limited possibilities and therefore were not sufficiently comprehensive to cover possible real-life instructed lying scenarios. To address these issues, this study employed a i) frequency domain network and connectivity analysis in the source space and ii) an original paradigm to induce subjects to fabricate lies about non-experienced events.

## Materials and Methods

### 2.1 Participants

Sixteen right-handed undergraduate students (8 males, 8 females) with a mean age of 22.5 years (SD = 4.5) were recruited as the subjects for this study. All of the subjects filled out a detailed health questionnaire, and their hearing ability and English proficiency were evaluated to ensure their suitability for participation. Only health-eligible subjects with satisfactory English proficiency were selected to participate. Each subject was given a detailed explanation of the experimental procedure, and each signed a consent form prior to the experiment. The study was approved by the NUS Institutional Review Board.

### 2.2 Deception task

The experiment contained four blocks, among which two belong to the WE task (instructed lying: IL, instructed truth: IT), which are related to autobiographic declarative/episode questions. The other two blocks belong to the NE task, which require subjects to fabricate lies for non-experienced events (refer to S1 Table and S2 Table for examples of stimulus). The four blocks were separated by 10-min breaks. The sequences of the four blocks were randomized across all of the subjects, and the sequences of the questions were randomized within the block. The question pool with 50 questions/condition were pre-screened by 15 volunteers (non-subject undergraduate students with similar population characteristic as the subjects) to score the understandability of each question into 4 levels (i.e. very easy-1, easy-3, moderate-difficult-5, very difficult-7). Understandability is a subjective score that measures how difficult it is for the volunteers to understand a particular question by asking the question once with the same speech rate. Examples were provided in advance as a customized calibration for each subject to know which level a particular question should fall into. After ranking the questions according to the understandability score, 25 questions with the lowest mean score were selected for each condition. In fact, the selected questions have been tested non-significantly different between the IL and IT in understandability (paired t-test: p = 0.75 for WE, and p = 0.82 for NE) and question length (paired t-test: p = 0.78 for WE, and p = 0.76 for NE) with the short-listed questions sharing a similar load between 2.2s-2.7s. In addition, prior to the experiment, subjects were instructed to give up the trial during the experiment by pressing a specific button if they cannot understand the question correctly. Truthful answers for all of the questions in the WE task were acquired in a pre-experiment investigation one week prior to the experiment for monitoring purposes during the experiment. The NE task mimics an interview scenario in which the questions are related to working experience in a target field (i.e., oil exploration) where none of the participants had prior experience. A pre-experiment survey was conducted on the spot to ensure that all subjects knew the terms that would be covered during the ‘mock interview’, and the subjects were instructed to pretend to be an experienced job candidate in the instructed lying condition when answering the questions for which they had no experiential knowledge.

**Figure 1:**
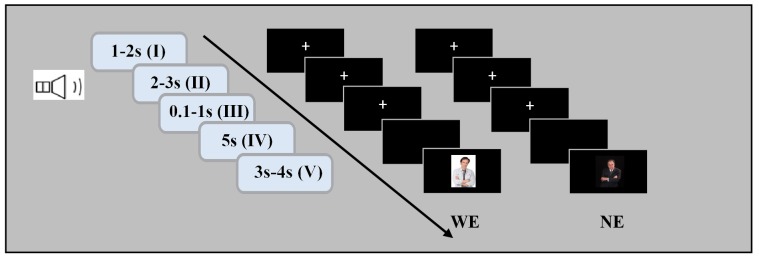
The WE and NE task for both the IL and IT conditions share the same schematic procedures. (I) fixation phase; (II) attending to the auditory question; (III) answering phase; (IV) mentalization phase; and (V) feedback phase, with differences in the visual images accompanying the auditory feedback for the two tasks to match the specific contexts, i.e. WE/NE.

The procedure in each block followed the same sequence (see Fig. 1). Each trial was initiated with a fixation period (3 s), after which an auditory stimulus was delivered to the subjects for 2.2–2.7s. After the subjects provided an answer to the questions, a ‘mentalization’ phase lasting for 5 s was added. This was triggered manually in the experiment by attending to the subjects’ verbalizations indicating the completion of an answer. The brain activities during this phase are not within the scope of this paper. After this phase, an auditory judgmental sentence with a varying degree of trust (i.e., believe/don’t believe/suspicious) was delivered to the subject and was simultaneously accompanied by a visual image of an interrogator in order to enhance the subject’s sense of reality in the experimental environment. The judgment was converted to an accumulated score with a deduction of 10 points for a judgment of lying, a deduction of 5 points for a judgment of a suspicious answer, and an addition of 10 points for the judgment of a lie as the truth. All updates were presented at the end of each trial, but the accumulated score was not reported to the subjects until the end of the experiment to avoid direct emotional interference imposed by the current score during the experiment. The ISI (inter-stimulus interval) varied from 3000 ms to 3500 ms; this length was used to avoid emotional effects caused by the procedure of the previous trial [13].

### 2.3 Instruction protocol

The subjects were informed prior to the experiment that they would be given a maximum of $50 once they successfully finished all of the blocks and that the actual reward would be adjusted based on the score achieved. However, if the frequency of the detected lies reached a pre-determined value in the lying block, they would receive only $25. This financial punishment helps to prevent low attention levels and effortless lying by the subjects. The subjects were required to answer as quickly and concisely as possible, but not to compromise the quality of the answer. To further engage the subjects’ attention, they were informed that each answer was judged by an experienced interrogator who did not know if they would tell lie or truth beforehand and monitored the answers in another room by receiving video and audio information from the experiment room. However, the actual judgment was manipulated based on a decision made by the experiment operator in the experiment room who knew the truthful answers and was monitoring the experiment via video and the verbal responses from the subjects. Additionally, the decision strategy for the judgment was integrated with a random effect to produce a 40% to 50% (true positive) chance of detection when the subjects were lying, and the misjudgment of truthful answers as lies was maintained below 20% (false positive). Feedback being used in this experiment is mainly because that the type of lying tasks (instructed lying) we are investigating intrinsically involves incentives and potential risk. Without such feedbacks, subjects’ perception of their ongoing behavior (i.e. lying/telling-truth) and the relevant awareness of being a liar/innocent would be weakened since their action is not associated with any consequence. The numbers picked up for chance of detection and false positive rate mainly serve the purpose to make the interrogator ‘appears’ with reasonably overall accuracy, and therefore helps maintain subject’s realistic feeling with the role of the interrogator and maintain their attention focus on the ongoing task. Despite this specific purpose, these figures are not critical parameters in this study due to the fact that EEG data within only the first 2s during the SDP were analyzed. Furthermore, this detection rule was not informed to the subject. Instead, subjects were informed that each feedback was given based on the information captured (e.g. video and verbal response) during the particular trial. To familiarize the subjects with the experiment procedure, they were provided with a training session with 10 sample questions for each condition, and they were required to repeat the rules to the operator to ensure that they fully understood all of the instructions.

### 2.4 Data acquisition and pre-processing

EEG signals were collected with an EEProbe recording system (Advanced Neuro Technology (ANT) Enschede, The Netherlands). The EEG cap with 64 Ag/AgCl electrodes was placed on each participant’s scalp according to the 10/10 system of electrode placement. Impedances for over 98% of the electrodes were guaranteed below 20 kOhm for each subject [14]. The sampling frequency was 250 Hz, and the common average served as the recording reference [15]. Presentation software (Neurobehavioral Systems, Inc., Albany, CA) was used to control the timing of the events in each trial. The auditory stimuli were prepared prior to the experiment using Ivona Reader software (IVONA Software, Poland), which transforms text information to standard auditory stimuli that were delivered to the participants through an earphone. To minimize the variation between the stimuli, the speed and intensity of the auditory stimuli were constant for all of the questions. During the experiment, the participants were comfortably seated in a quiet room with a controlled temperature of 25°C. A computer screen was displayed in front of the subject with a fixed viewing distance of 80 cm. A webcam was mounted on the top of the computer screen for online video recording, which enabled the experiment operator to provide a trial-based decision to the trial. This operation could not be detected by the participants. An electromyography (EMG) signal was simultaneously recorded with an electrode that was attached to the subject’s face, and the location was found not to interfere with speaking. This signal was used to determine the onset time of the verbal response. An electrooculography (EOG) signal was simultaneously recorded using two Ag/AgCl electrodes placed on the vertical midline of the right eye above the eyebrow and approximately 1 cm below the lower lid. In the off-line analysis, the EEG signals were band-pass filtered at 1–30 Hz. The EOG and EMG artifacts of the collected EEG data were removed using open-source artifact removal software [16], with the application of independent component analysis (ICA) algorithms, i.e. canonical correlation analysis(CCA) as a blind source separation technique for EMG removal [17] and sobi for EOG removal [18] for each individual trial. Since band pass filtering, EOG and EMG removal were applied simultaneously to de-noise the data, only a few trials still produce noisy data after de-noising (might be caused by subject’s body motion at some trials) and have to be discarded. Thus, a minimum of 22 and a maximum of 25 valid trials were collected for each condition (condition WE-IL: mean: 24.43, variance: 1.06; condition WE-IT: mean: 24.50, variance: 0.80; condition NE-IL: mean:24.44, variance: 0.80; condition NE-IT: mean: 24.50, variance: 0.53), which approximates the starting trial number (i.e. 25) for time-frequency analysis as suggested by [19]. Moreover, this study adopted frequency domain connectivity analysis, with 2s per trial altogether providing 44–50s data length per subject, which should render reasonably accurate estimation with the employed estimator given the fair SNR after the de-noising procedures [20].

### 2.5 Data analysis

**Figure 2:**
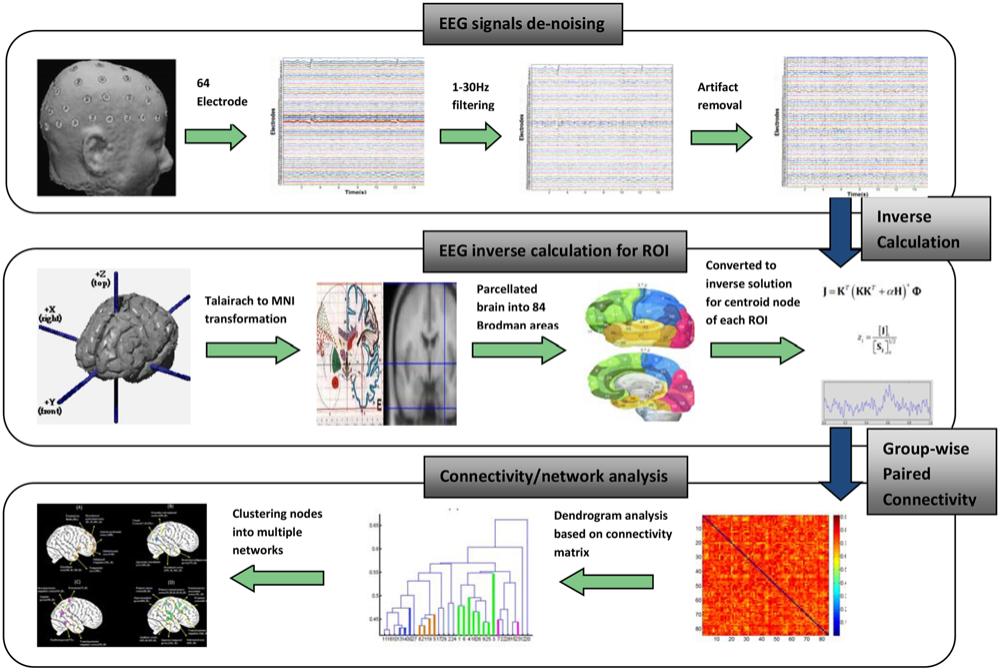
The descriptive steps of analysis from input EEG raw signal to the output network. The ‘EEG-signal’ segment dealt with band-pass filtering (1–30Hz) as well as artifact (EOG & EMG) removal of raw EEG data (output ‘clean’ EEG data); the ‘EEG inverse calculation for ROI’ mainly mapped the ‘clean’ EEG into source space on centroid voxel that is most representative of the 84 ROIs to retrieve source time series; the pair-wise connectivity was calculated based on the 84 ROI’s time series into a 84×84 matrix, and then a group-wise connectivity matrix underwent dendrogram analysis that clusters 84 nodes into groups of sources, among which 4 common networks for the IL and IT conditions were identified and mapped onto a generic cortex model.

The experimental modulation effects on the inter-regional neural activities were analyzed through frequency domain connectivity analysis [21–24]. A network analysis was performed based on the connectivity computation and dendrogram analysis, which enables the identification of common sub-networks underlying various experimental conditions [25]. The dendrogram clustering analysis used average linkage clustering based on correlation distance [26]. Among the various types of connectivity measurements, phase-lagged index (PLI) [27], lagged-phase synchronization (LPS) [28], partial-directed coherence (PDC) [29], and granger causality (GC) [30] are frequently employed. In this study, LPS was applied for connectivity measurements because this method provides zero-lag removal connectivity, which largely mitigates the contamination issue caused by volume conduction that is intrinsically present in EEG recordings either on the scalp or at the source level. Moreover, we were interested in the connectivity changes that were modulated by the experimental effects irrespective of the exact interactive direction. In the connectivity analysis, the absolute and event-related connectivity were calculated for the first 2s time window during the phase of SDP on the cerebral cortex, which is segmented into 84 Brodmann (BA) areas (i.e. 42 BAs each hemisphere) based on the cytoarchitectonics of neurons [31], and the LPS is defined according to Pascual-Marqui RD et al. [28]. Additionally, the clustering coefficient was computed based on the definition according to Chu CJ et al. [32], which generally characterizes the node’s interaction with the rest of the brain. Normalization procedure was performed on connectivity and clustering coefficient measurement with respect to those measured from the baseline window (i.e. the last 2s of the baseline segment) to remove the different baseline effects for different conditions. As such, the connectivity/clustering coefficient derived after normalization is called event-related connectivity/clustering coefficient. Standardized low-resolution brain electromagnetic tomography (sLORETA) [33] was used to compute the connectivity in selected frequency bands (i.e., the upper alpha band) with a source solution space consisting of 6,239 voxels (voxel dimensions 5×5×5 mm) that was restricted to the cortical gray matter and hippocampi as defined according to the MNI atlas [34]. As recommended by sLORETA, single voxel at the ROI centroid was selected for connectivity calculation for two reasons: (1) The calculation of source by sLORETA is based on the assumption that the smoothest of all possible activation distributions is the most plausible one, and this is supported by neuro-physiological data demonstrating that neighbouring neuronal populations present highly correlated activity [35]. Due to this assumption, signals of spatially adjacent voxels of neighbouring ROIs are highly correlated, inducing larger connectivity, which might not be physiological in nature. By taking this single centre voxel of each ROI, such contamination for connectivity estimation could be minimized, since information of the centroid voxel is an accurate representative for activity within the ROIs [36]. (2) If you define a large ROI, then the average sLORETA activity spanning a large volume might not be very meaningful. Therefore, given the low spatial resolution of sLORETA, computation for the centroid voxel was chosen and as a result, the network analysis is not refined but mainly captures the dominant features of connection. The cortical connectivity estimation is then based on the electrical current source time series estimated by sLORETA [36]. In the network analysis, a dendrogram that consisted of mirrored C-shape lines was employed to classify the 84 ROIs into a hierarchical cluster tree according to their proximity. Based on the distance calculation for all of the paired nodes (ROIs), which is defined as ‘1-connectivity’ and is opposite the trend of connectivity, a dendrogram was created that enables the clustering of nodes into a sub-network sharing a similar connection pattern [25] with respect to the remaining brain regions. The dendrogram analysis was performed based on the averaged connectivity matrix across the subjects that provides a group-wise connectivity matrix for each condition [25, 36], and the average procedure is assumed to improve the SNR for the estimation of the connectivity matrix and helps facilitating the identification of the most common and stable network patterns across subjects. Possible concerns regarding the connectivity/functional network analysis are 1) deception is a complicated process which involves multiple known or unknown cognitive/sensory processes, and 2) the analysis window where talking is involved or too significant time variation is involved (i.e. between the offset of stimuli and the onset of the answering) does not provide reliable and consistent EEG signal due to too much EMG artifact. Luckily, the dendrogram analysis was only performed for a narrow window (i.e. the first 2s of stimuli delivery) bearing more or less predictable underlying sub-processes. Finally, behavior data (response time) were extracted based on the filtered EMG signal (band-pass: 1–25 Hz), which was recorded from a separate EMG channel for correlation analysis with network-scale neuronal activities. In this study, multiple comparison permutation tests were widely employed for all the statistical comparisons on the analyzed index between conditions or correlation analysis based on [37] and [38]. The multiple comparison permutation paired test employs a ‘tmax’ method for paired comparisons which adjusts the p-values of each variable for multiple comparisons among all the 84 ROIs (corrected p<0.05). The multiple comparison permutation correlation tests is based on Spearman’s rank correlation coefficient (ρ).When applying the test to multiple variables, the ‘max statistic’ method is used for adjusting the p-values of each variable for multiple comparisons among all the 84 ROIs (corrected p<0.05). Like Bonferroni correction, this method adjusts p-values in a way that controls the family-wise error rate. However, the permutation method will be more powerful than Bonferroni correction when different variables in the test are correlated. Technically, all the multiple comparison corrected t-tests in this study were performed based on open-source Matlab codes [39, 40]. The steps of analysis from input EEG data to output network have been illustrated in Fig. 2.

## Results

### 3.1 Network analysis

In the functional network analysis, the connectivity between regions was calculated based on the un-normalized LPS index because components of the network during the first 2s time window of SDP is independent of the baseline window. A data-driven dendrogram analysis was performed based on the connectivity matrix which represents all nodes’ connection pattern with the rest of the brain for both the IL and IT conditions in either task. From these components, four groups were qualitatively selected based on the dendrogram and visual inspection and this method has been applied in a previous study [25]; additionally, the sub-networks are required to potentially depict functionally related groups in both the IL and IT conditions.

**Figure 3:**
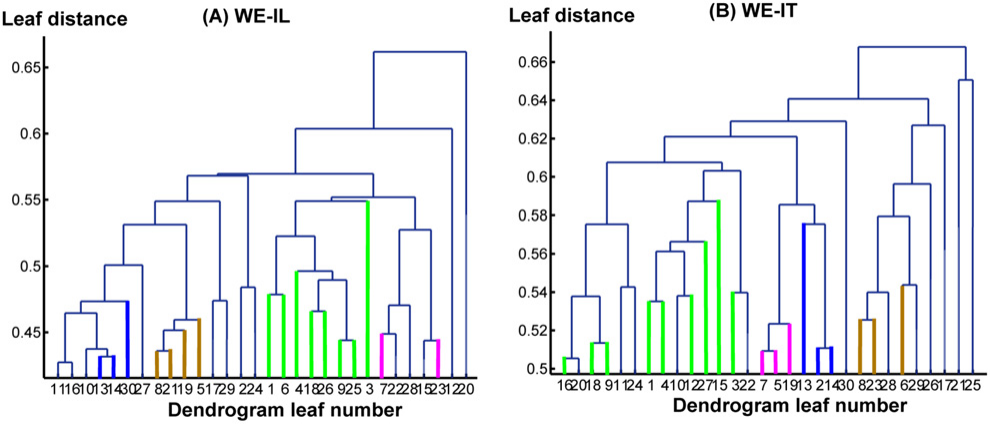
The hierarchy structures based on the dendrogram analysis for the WE-IL (A) and the WE-IT (B) conditions, respectively. The x-axis: leaf number, with each leaf representing a cluster of BAs. The y-axis represents leaf distance calculated using average linkage clustering based on correlation distance. The orange color indicates group A, the blue color indicates group B, the pink color indicates group C, and the green color indicates group D.

**Figure 4:**
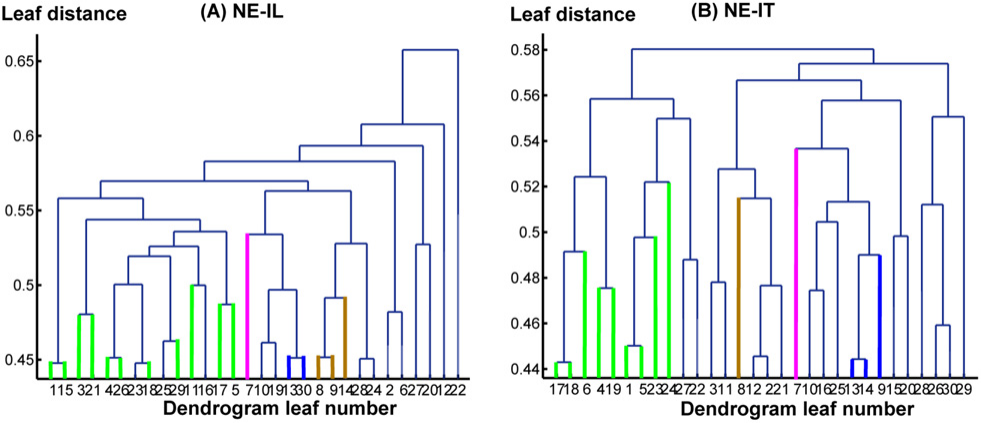
The hierarchy structures based on the dendrogram analysis for the NE-IL (A) and the NE-IT (B) conditions, respectively. The x-axis: leaf number, with each leaf representing a cluster of BAs. The y-axis represents leaf distance calculated using average linkage clustering based on correlation distance. The orange color indicates group A, the blue color indicates group B, the pink color indicates group C, and the green color indicates group D.

**Figure 5:**
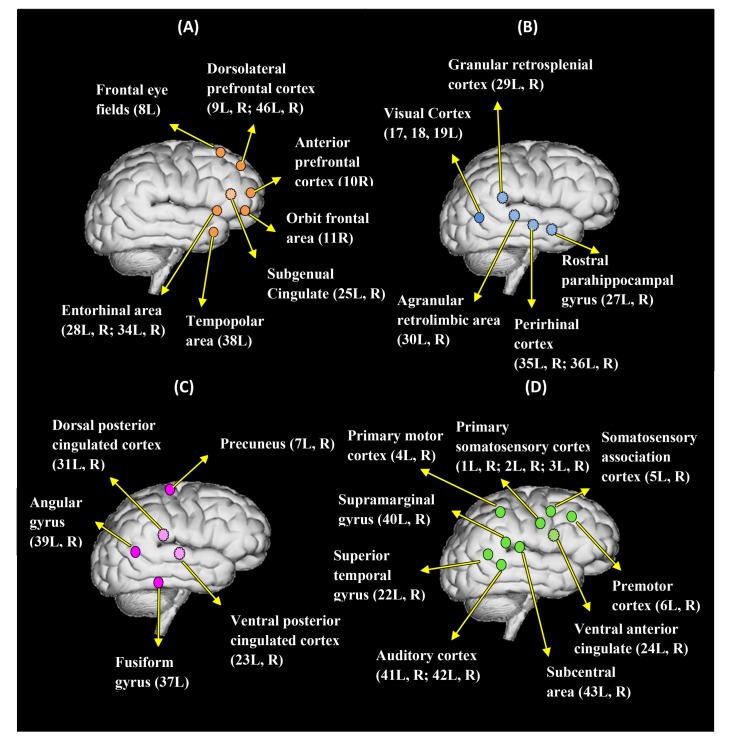
The four common sub-networks (functional clusters) for the WE-IL and the WE-IT conditions. (A)-(D) are hypothetical networks for executing higher-order function, comprehension/memory processing, and speech perception, respectively. The dotted circle indicates a medial surface source, while the solid circle indicates a lateral surface source. The label for each anatomical region includes the Brodmann area name and the laterality information.

**Figure 6:**
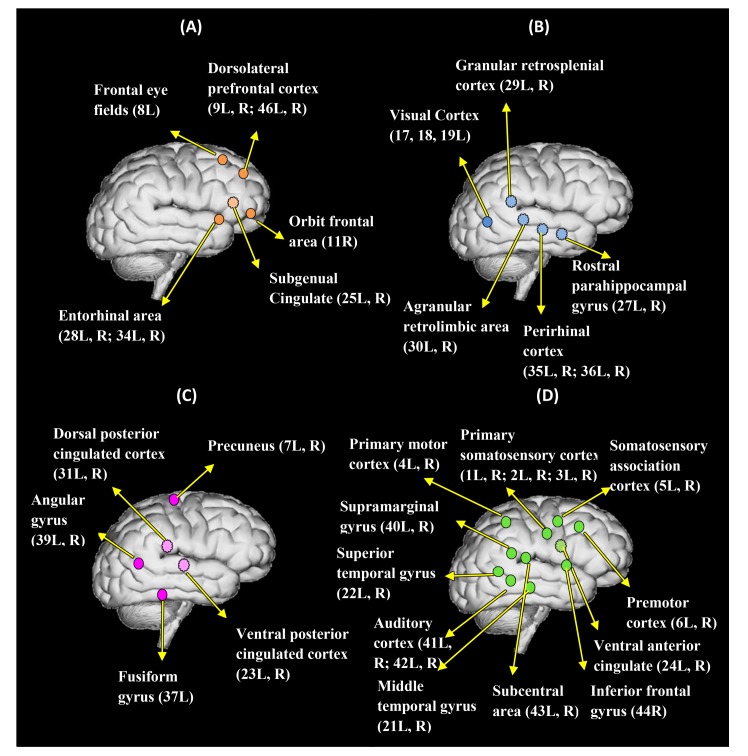
The four common sub-networks (functional clusters) for the NE-IL and the NE-IT conditions. (A)-(D) are hypothetical networks for executing higher-order function, comprehension/memory processing, and speech perception, respectively. The dotted circle indicates a medial surface source, while the solid circle indicates a lateral surface source. The label for each anatomical region includes the Brodmann area name and the laterality information.

Interestingly, several sub-networks were identified that were shared by both the IL and IT conditions in both tasks. The dendrogram analysis results are depicted in Fig. 3 and Fig. 4 for the WE and the NE tasks, respectively, where Fig. 3 and Fig. 4 summarize the information of the connectivity matrix for all of the cortical areas in each condition into a dendrogram by clustering nodes that share similar connection patterns into sub-networks. In Fig. 3 and Fig. 4, different colors are used to indicate different types of sub-networks. Corresponding sources for each sub-network are plotted on a cortical brain surface (as shown in Fig. 5 and Fig. 6) with the corresponding color used in Fig. 3 and Fig. 4. The detailed sub-network source anatomical information is described in Table 1 and Table 2 for the WE and the NE tasks, respectively.

**Table 1:** Results from the dendrogram analysis for the WE task; four groups of sources were found to be common to the WE-IL and the WE-IT conditions in the upper alpha band.

**Group A**	frontal eye fields (8L), dorsolateral prefrontal cortex (9L, 9R, 46L, 46R), anterior prefrontal cortex (10R), orbitofrontal area (11R), subgenual cingulated (25L, 25R), entorhinal area (28L, 34L), entorhinal area (28R, 34R), temporopolar area (38L)
**Group B**	visual cortex (17L, 18L, 19L), rostral parahippocampal gyrus (27L, 27R), granular retrosplenial cortex (29L, 29R), agranular retrolimbic area (30L, 30R), perirhinal cortex (35L, 36L, 35R, 36R)
**Group C**	precuneus (7L, 7R), posterior cingulate cortex (23L, 23R), dorsal posterior cingulate area (31L, 31R), occipitotemporal area (37L), angular area (39L, 39R)
**Group D**	primary somatosensory cortex (1L, 1R, 2L, 2R, 3L, 3R), primary motor cortex (4L, 4R), somatosensory association cortex (5L, 5R), premotor cortex (6L, 6R), ventral anterior cingulate area (24L, 24R), supramarginal gyrus (40L, 40R), subcentral area (43L, 43R), superior temporal gyrus (22L, 22R), auditory cortex (41L, 41R, 42L, 42R)

**Table 2:** Results from dendrogram analysis for the NE task; four groups of sources were found to be common to the NE-IL and the NE-IT conditions in the upper alpha band.

**Group A**	frontal eye fields (8L), dorsolateral prefrontal cortex (9L, 46L, 9R, 46R), entorhinal area (28L, 34L), entorhinal area (28R, 34R), subgenual cingulated (25L, 25R), orbital area (47L)
**Group B**	visual cortex (17L, 18L, 19L), rostral parahippocampal gyrus (27L, 27R), granular retrosplenial cortex (29L, 29R), agranular retrolimbic area (30L, 30R), perirhinal cortex (35L, 36L, 35R, 36R)
**Group C**	precuneus (7L, 7R), ventral posterior cingulate cortex (23L, 23R), dorsal posterior cingulate cortex (31L, 31R), occipitotemporal area (37L), angular gyrus (39L, 39R)
**Group D**	primary somatosensory cortex (1L, 1R, 2L, 2R), primary motor cortex (4L, 4R), somatosensory association cortex (5L, 5R), premotor cortex (6L, 6R), ventral anterior cingulate cortex (24L, 24R), supramarginal gyrus (40L, 40R), subcentral area (43L, 43R), middle temporal gyrus (21L), superior temporal gyrus (22L, R), auditory cortex (41L, 42L, 41R, 42R), inferior frontal gyrus (44R)

Despite the similarities in sub-network components shared by the two conditions in the WE task, differences were found via further analyses. In Table 3, the inter- and intra-group connectivities were compared between the WE-IL and the WE-IT conditions. The intra-group connectivity/inter-group connectivity was calculated based on the summation of all of the non-repeated paired connectivity values between regions that belonged to the two inspected groups, respectively. During the calculation, two indexes that could potentially represent the inter- and intra-group connectivities were calculated for the first 2s window of the auditory stimuli. The first index, c1, is a normalized connectivity value that indicates the percentage of change for connectivity in the SDP relative to that of the baseline period, i.e., the last two seconds of the ‘fixation’ phase. To some extent, this measurement helps to exclude spurious connection at a statistical level based on how relevant the connection is to the task (i.e. the lower the absolute value, the more irrelevant to the task, and hence less likely to present significant differences between conditions). As a result, the c1 index measures the degree of event related modulation on within- and between- network connectivity by the event (tasks during the SDP window). The other index, c2, is an un-normalized absolute connectivity value for the first 2s during the SDP window. As referenced in Table 2, the intra-group connectivity does not show any significant difference between the IL and the IT conditions, while the inter-group connectivity shows a significant increase in the IL condition relative to the IT condition in multiple group pairs, i.e., group A↔B, group A↔C, group A↔D, group B↔D, and group C↔D by c1 index after multiple comparison permutation tests (corrected p<0.05) for inter- comparisons (four networks’ comparisons) and intra- comparisons (six pairs’ comparisons) separately. In addition, as predicted, c1 is more sensitive than c2 for representation of significant differences in inter-group connectivity between the IL and IT conditions.

**Table 3:** The difference in the intra- and inter-group connectivity among sub-networks in the upper alpha band between the WE-IL and the WE-IT conditions.

**Intra-Group Connectivity Increase**		**Inter-Group Connectivity Increase**	
**Group name**	**p-Values for c1 and c2**	**Group pairs**	**p-Values for c1 and c2**
**Group A**		Group A ↔ Group B	↑(p1 = 0.0177); −(p2 = 0.1030)
	−(p1 = 0.0657, p2 = 0.1146)*	Group A ↔ Group C	↑(p1 = 0.0333); −(p2 = 0.1304)
		Group A ↔ Group D	↑(p1 = 0.0150); −(p2 = 0.1892)
**Group B**	−(p1 = 0.1206, p2 = 0.0989)	Group B ↔ Group C	−(p1 = 0.3907, p2 = 0.2204)
		Group B ↔ Group D	↑(p1 = 0.0262); −(p2 = 0.2220)
**Group C**	−(p1 = 0.2047, p2 = 0.1436)	Group C ↔ Group D	↑(p1 = 0.009); −(p2 = 0.1128)
**Group D**	−(p1 = 0.0945, p2 = 0.2741)		

*p1and p2 are corrected p-values after multiple comparisons permutation test for c1 and c2 between the IL and IT conditions, respectively

The sources of groups A, B, C, D are depicted in Fig. 5.

Correspondingly, in Table 4, the inter- and intra-group connectivities were compared between the NE-IL and the NE-IT conditions with multiple comparison permutation tests (corrected p<0.05) for inter- comparisons (four networks’ comparisons) and intra- comparisons (six pairs’ comparisons) separately. Interestingly, group A showed decreased intra-group connectivity by c2 index, while group C showed increased intra-group connectivity by c1 index. The inter-group connectivity increased in two group pairs, i.e., group A↔D and group B↔C by c1 index.

**Table 4:** The difference in the intra- and inter-group connectivity among sub-networks in the upper alpha band between the NE-IL and the NE-IT conditions.

**Intra-group Connectivity Increase**		**Inter-group Connectivity Increase**	
**Group name**	**p-Values for c1 and c2**	**Group pairs**	**p-Values for c1 and c2**
**Group A**		Group A ↔ Group B	−(p1 = 0.1558, p2 = 0.5137)
	−(p1 = 0.4870, ↓p2 = 0.0356)*	Group A ↔ Group C	−(p1 = 0.1589, p2 = 0.6420)
		Group A ↔ Group D	↑(p1 = 0.0275, p2 = 0.2209)
**Group B**	−(p1 = 0.2464, p2 = 0.4444)	Group B ↔ Group C	↑(p1 = 0.0175, p2 = 0.4732)
		Group B ↔ Group D	−(p1 = 0.1072, p2 = 0.5699)
**Group C**	↑(p1 = 0.0293, p2 = 0.3993)	Group C ↔ Group D	−(p1 = 0.1200, p2 = 0.4775)
**Group D**	−(p1 = 0.4259, p2 = 0.2972)		

*p1and p2 are corrected p-values after multiple comparisons permutation test for c1 and c2 between the IL and IT conditions, respectively

The sources of groups A, B, C, and D are depicted in Fig. 6.

### 3.2 Connectivity analysis

A connectivity analysis was performed for all possible pairs of 84 Brodmann areas which give rise to a connectivity matrix. Based on which, event-related clustering coefficient was derived for each node that characterizes the node’s interaction with the rest of the brain under modulation by the stimulus. Table 5 and Table 6 show the ROIs whose event-related clustering coefficients significantly increased in the upper alpha band in the WE-IL compared with the WE-IT condition (Table 5) and in the NE-IL compared with the NE-IT condition (Table 6) after multiple comparison permutation test among all the 84 ROIs with corrected p<0.05.

**Table 5:** Source level clustering coefficients increase in the upper alpha band in the WE-IL relative to the WE-IT condition (after multiple comparison permutation tests with corrected p<0.05).

**Functional Name**	**BA/hemisphere**	**Functional Name**	**BA/hemisphere**
dorsolateral prefrontal cortex	9L	pregenual cortex	33L, 33R
retrosplenial cingulated cortex	29L	perirhinal cortex	35L, 36L
visual cortex	19L	primary motor cortex	4R
parahippocampal gyrus	27L	inferior temporal cortex	20R
entorhinal cortex	28L, 34L		

**Table 6:** Source level clustering coefficients increase in the upper alpha band in the NE-IL relative to the NE-IT condition (after multiple comparison permutation tests with corrected p<0.05).

**Functional Name**	**BA/hemisphere**	**Functional Name**	**BA/hemisphere**
visual cortex	19 L, 17R	subgenual cingulated	25 L, 25R
posterior insular cortex	13 L, 13R	granular retrolimbic area	30 L, 30R
superior temporal gyrus	22 L	perirhinal area	35 L
entorhinal area	34 L	posterior transverse temporal area	42 L
subgenual area	43 L	postcentral area	3R
premotor cortex, SMA	6R	orbital area	11R
angular gyrus	39R		

### 3.3 Response time (correlation) analysis

**Figure 7:**
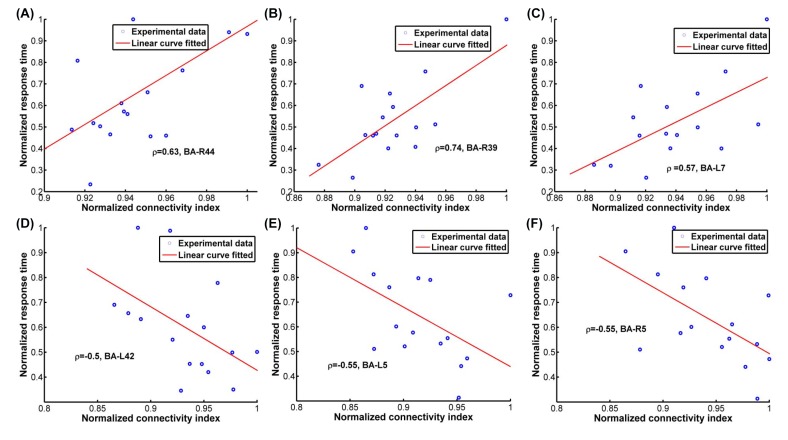
Several regions’ normalized event-related clustering coefficients were found positively correlated with the normalized response time in different conditions. The event-related clustering coefficient (normalized) of the inferior frontal gyrus (a) was found to be positively correlated with the response time (normalized) in the WE-IL condition (corrected p<0.05). The event-related clustering coefficients of the angular gyrus (b) and precuneus (c) were found to be positively correlated with the response time (normalized) in the WE-IT condition (corrected p<0.05). The event-related clustering coefficient (normalized) of the secondary auditory cortex (42L) (d) was found to be marginally negatively correlated with the response time (normalized) in the NE-IL condition (corrected p = 0.058). The event-related clustering coefficient of the somatosensory association cortex (L, R) (e, f) was found to be negatively correlated with the response time (normalized) in the NE-IT condition (corrected p<0.05). All the significance reports are based on multiple comparison permutation correlation test with corrected p<0.05.

Because the response time of the subject’s answer to each question is closely related to the overall processing time spent by all of the involved sub-networks and the key processing nodes in the brain, it can be hypothesized that there may be a quantitative relationship (i.e., linear/non-linear) between the response time and the activity of the sub-networks or key nodes that contributed dominantly to the variation in processing time. To study this possible relationship, a correlation analysis was performed between the response time and the clustering coefficient of each cortical area which characterizes the node’s interaction with the rest of the brain (Fig. 7). The rationale for this is two-fold: i) correlation is a method to study linear relation, while nonlinear relation is more sophisticated and less easily clarified, and ii) as mentioned earlier, all nodes in a specific sub-network share a similar connection pattern with the rest of the brain. Therefore, each node can to some degree be representative of its affiliated sub-network’s connection pattern. Although the second consideration has not excluded the correlated behavior of all of the nodes affiliated with the same sub-network, it is sufficient to help identify the potential correlation between response time and the underlying sub-network activity. However, the second consideration is not an issue for the key processing node, which may not be affiliated with a specific network.

A correlation analysis was performed via linear regression analysis, which tests the significance of the positive/negative correlation and produces a linear correlation coefficient. Two types of data set were used in the linear regression analysis, i.e., the normalized average response time for each subject in each condition and the normalized average clustering coefficient for each subject in each condition. Averaging was performed across the trials in each condition for the response time and the clustering coefficient to minimize the noise level for the correlation analysis. Normalization was performed for the averaged response time and the event-related clustering coefficient to fall in [0,1] which makes the linear correlation coefficients fall between 0 and 1 as a standard range. As referenced in the results (Fig. 7), several regions were found to be positively/negatively correlated with the normalized response time. Specifically, the normalized event-related clustering coefficient for the inferior frontal gyrus (44R) was found positively correlated with the normalized response time in the WE-IL condition (p<0.05, with multiple comparison correction [38]), and the normalized event-related clustering coefficient of the angular gyrus (39R) and precuneus (7L) was found to be correlated with the normalized response time in the WE-IT condition (p<0.05, with multiple comparison correction [38]). However, the normalized event-related clustering coefficient of the secondary auditory cortex (42L) was found to be marginally negatively correlated with the normalized response time in the NE-IL condition (p = 0.058), while that of the somatosensory association cortex (5L, R) was found to be negatively correlated with the normalized response time in the NE-IT condition (p<0.05, with multiple comparison correction [38]).

## Discussion

### 4.1 Network analysis

The results of the network analysis are presented in Figs. 3–6 and Tables 1–4. It should be noted that the identified sub-networks may be part of a larger hierarchical network. However, because the focus is to identify the common networks shared among the IL and the IT conditions during the SDP, our discussion may not cover the complete network involved in each condition. Due to the limited length of window analyzed, i.e. the first 2s during SDP, in general, it is most likely that the neuropsychological processes involved are confined to those related with auditory stimulus perception, comprehension of the question, memory recall based on the question being asked, and overall executive control over multiple simultaneous processes. It is no doubt that auditory stimulus perception is a sentinel and must-have component throughout the whole time window. Upon which, comprehension is established through interaction of processing on the perceptual input and retrieval of stored memories, i.e. declarative memory and episodic memory. As such, higher-order executive control unit must be recruited in order to monitor the ongoing processes by integration of input information and navigating the response (i.e. deceptive/truthful) to the question. Most importantly, these are the networks shared by both the IL and the IT conditions. Although the analyzed window has not covered the deception window directly, this analysis provides a unique perspective from stimulus delivery phase during which there may exist commonalities and differences between conditions as potential features. This narrow window chosen for network analysis bearing more or less predictable underlying sub-processes may potentially initiate an exciting research direction seeking for systematic level of understanding beyond the component-based perspective (i.e. inhibition control, saliency detection, risk monitoring) to complement the deception insights.

Among the sub-networks identified for the WE task (Fig. 3, 5; Tables 1, 3), it can be postulated that group A may serve as a higher-order executive control network as inferred from the network’s neuro-anatomy. Due to the fact that dorsolateral prefrontal cortex (DLPFC), anterior prefrontal cortex (APFC), orbitofrontal area and entorhinal areas are undisputed cortical regions with higher-order functions [41, 42], it is most likely they synergistically contribute to manage and support other simultaneous cognitive and sensory functions during the time window analyzed. Dorsolateral prefrontal cortex (DLPFC) has been implicated in many higher-order functions, including holding spatial information ‘on-line’, response selection, verification and evaluation of representations that have been retrieved from long-term memory, monitoring and manipulation with working memory [41]. In addition, Anterior prefrontal cortex (APFC) has been engaged with a unified role in coordination of information processing and information transfer between multiple operations across supramodal cortex when problems involve more than one discrete cognitive process [41]. Orbitofrontal area has been implicated in processes that involve motivational/emotional value of incoming information [43], the representation of learnt relationships between arbitrary stimuli and reward/punishment, and integration of these information to guide response, decision making [41]. Entorhinal area has been considered as a hub functioning in a widespread network for memory and navigation [44]. This cortex, in conjunction with the hippocampal formation, appears to specifically deal with the translation of neocortical exteroceptive information into higher order complex representations that, when combined with motivational and interoceptive representations, serves cognitive functions, in particular conscious memory and relational organization of memory [45]. Compared with the above regions, subgenual cingulated has less higher order role but has been demonstrated to play an integral role in both normal and pathological shifts in mood [46] and processing of positively or negatively valenced stimuli [47]. As such, it can be seen that most of the above regions are well postulated to underlie a network executing ‘higher-order’ functions that support and manage multiple simultaneous processes during the task.

Given the neuro-anatomy of group B, it can be inferred that this group may contribute to multi-modal contextual association and object recognition in support of comprehension and memory processing. The neuro-anatomy seems to infer the involvement of the ventral stream pathway. This processing pathway usually begins purely with visual information in the primary visual cortex (occipital lobe), and then this information is transferred to the temporal lobe. This pathway is mainly involved in object recognition (i.e. the ‘what’ pathway), and is connected to the medial temporal lobe (which is involved in the storage of long-term memories) [48]. Although no visual stimulus has been used in the experiment, visual cortex can be activated by visual imagery of the objects contained in the stimulus [49] considering the fact that most of the stimulus tend to induce declarative and episodic memory with requirement to recall specific objects. Not surprisingly, temporal regions such as rostral parahippocampal gyrus and perirhinal cortex (PC) involve in this network. Previous studies have shown perirhinal cortex plays an important role in recognition memory by facilitating the recognition and identification of the stimuli through stimulus-stimulus (i.e., word-object) associations [50]. Rostral parahippocampal gyrus as a portion of parahippocampal gyrus, located at the medial temporal lobe, is a key structure in declarative and episodic memory processing [51, 52]. Recently, a broad synthesis of literature has unified the various functions of this area by the theory of ‘contextual association’, i.e. to define, represent items by bringing meaning to the environment and establish links between these contextual items [51]. The granular retrosplenial cortex and agranular retrolimbic area have also been linked with cross-modal object recognition by observing rats with lesions in these regions performing cross-modal object recognition task [53], hence suggesting their role in mediating the integration of information across multiple cue types.

Similar to group B, group C is inferred to be participating as a portion of network to support the comprehension and memory-processing processes. It has been suggested in previous studies that the anterior medial parietal/posterior cingulate cortex is concerned with linking information with prior knowledge [54], and has repeatedly been associated with episodic memory retrieval [55]. Previous studies have found that precuneus is involved in a wide spectrum of highly integrated tasks, including visuo-spatial imagery, episodic memory retrieval and self-processing operations, namely first-person perspective taking and an experience of agency [56]. Studies have also shown that occipitotemporal area plays an important role in constructing scenes from objects by supporting recognition of real-world visual scenes through parallel analysis of within-scene objects [57]. Moreover, the angular gyrus has been considered as a cross-modal hub where converging multisensory information is combined and integrated to comprehend and give sense to events, manipulate mental representations, and reorient attention to relevant information [58]. It can be inferred further that these above regions play a central role to facilitate recognition of real-world visual scenes/events and give sense to these scenes/events through a first-person perspective. Compared with group C, group B seems to be more associated with function in establishing contextual association (i.e. word-object) and object recognition, and both contribute as part of a network that support comprehension and memory processing. Nevertheless, group B seems to be associated more with ventral stream pathway, and group C is more associated with dorsal stream pathway.

Different from the former three groups, group D involves mostly perception related regions, and it is most likely a network contributing to auditory and language processing. Auditory cortex is a classic highly organized region that perceives sound, and this cortex area is the neural crux of hearing for humans in language and music [59]. Superior temporal gyrus has been associated with various functions, among which, the most likely function in this experiment is the auditory processing as a key sensory structure contributing to comprehension of language [60, 61]. The superior temporal gyrus known as Wernicke’s area is one of the human speech areas involved in the understanding of written and spoken language [62]. The Wernicke’s area in the non-dominant hemisphere is involved in processing and resolution of subordinate meanings of ambiguous words, while the counterpart in the dominant hemisphere is associated with processing dominant word meanings. In addition, previous fMRI study has found subcentral area’s involvement in semantic processing, and it is activated when participants are asked to complete tasks which involved the selection of a verbal response from many possible responses [63]. As an intrinsic and implicit requirement of our experiment, when subjects listen to the questions, their brains are engaged in looking for possible verbal response simultaneously. Involvement of the somatosensory system in the perceptual processing in speech is not clearly known until recent studies have shown that somatosensory input has modulation effect on speech perception [64, 65]. Similarly, involvement of motor areas such as primary motor cortex and premotor cortex in speech perception can be linked to the concept that perception and production are mediated by common mechanisms originates in the motor theory of speech [66, 67], evidence to date for the link between perception and production comes from demonstrations of cortical motor activation in conjunction with speech perception [68–70], and is related to articulating speaker’s gesture [70] or mapping articulatory features of speech sounds [71]. According to the neuro-anatomy for language/speech perception, part of parietal region is also involved. Specifically, supramarginal gyrus is thought to be probably involved with language perception and processing (e.g. in phonemic perception and the processing of speech sounds), since lesions in it may cause receptive aphasia [72, 73]. Primary somatosensory cortex and ventral anterior cingulated area are not typical regions associated with semantic processing; however, ventral anterior cingulated area has been related to self-referential processing of negative stimuli in semantic processing [74] and primary somatosensory cortex (postcentral gyrus) is involved in attentional and linguistic interactions in speech perception [75].

Although there is no nominal reference to a specific source and its definitive network category or vice versa, the data-driven functional network results obtained in this study are reasonably justifiable. It is not surprising that the sub-networks identified are neuro-psychological processes that appear to be necessarily involved during the analyzed window. Therefore, the top-down perspective of network analysis appears to match well with the data-driven networks.

In within- and between- network analysis, the results from Table 3 show that p1 has a greater significant difference compared with p2 while nearly maintaining the same trend of p2, which means that a normalized connectivity is potentially more sensitive than an un-normalized connectivity in distinguishing the two conditions in the WE task. An increased trend for inter-group connectivities was found for groups A; this increase is marginally significant (p<0.1) and could be interpreted as increased communicative activity within the higher-order executive control group that was induced by the cognitive load of deception. Significant increases were found among several pairs of groups in inter-group connectivity; connections significantly increased between i) group A↔group B, ii) group A↔group C, iii) group A↔group D, iv) group B↔group D and v) group C↔group D. As inferred on each network’s most likely functional role, group A as a higher-order executive area represents an extensive increased communicative activity with the parallel memory processing, comprehension, speech perception processes. This further implies the higher-order role of group A in the IL condition, and that the IL condition in the WE task potentially induces enhanced executive control activities, even during the stimulus delivery phase. Furthermore, increased connectivities between B and D, C and D imply that speech perception more closely interacts with comprehension and memory processing to facilitate the deception process.

A functional network analysis was also applied for the NE-IL and NE-IT conditions. The results are shown in Fig. 4, 6 and Table 2, 4. Again, four groups were identified as sub-networks. Interestingly, these functional networks are the exact sub-networks that were identified for the WE task with minor variation in the network component. As shown in Table 4, a significant decrease of intra-network connectivity has been observed based on the p2 index for group A in the NE-IL condition relative to the NE-IT condition. This is most likely because this group’s higher-order function could be less efficiently executed when the subjects had no prior experience related to the questions. But since this index is not normalized, this result is still arguable. In contrast, intra-network connectivity increased in group C for the p1 index possibly due to the increased load in comprehension and memory-recall of questions non-experienced by the subjects. However, significant increases in the inter-group connectivity based on the p1 index have been observed i) between group A↔group D and ii) group B↔group C. This means the interaction within network possibly responsible for memory processing and relevant comprehension processes increased, since group B and C both partially contribute as sub-network. In addition, connectivity between networks possibly related to executive control and speech perception increased, which implies speech perception for unfamiliar items may need increased cognitive input from executive network in order to make sure the auditory information can be stored well in short-term memory for accurate comprehension.

In summary, the application of dendrogram analysis enables us to identify the most likely fundamental common processing units shared by the WE and NE tasks. It can be observed that the formation of all of the groups in the NE task are very similar to the corresponding groups in the WE task, indicating the potential role of these sub-networks as fundamental building blocks for the instructed lying/truth-telling tasks that involve the processing of auditory questions; the robustness of this result has been cross-validated by the two tasks. However, this result does not exclude the possibility for additional sub-networks to participate in each of these tasks due to the fact that only the first 2s during the SDP window was analyzed. The network derivation has not yet been cross-validated in other studies; thus, it is important to re-affirm the interpretation of the functional role of these sub-networks via cross-validation with fMRI studies in the future.

### 4.2 Connectivity analysis

In a further analysis as shown in Table 5, multiple memory processing regions were found with an increased overall clustering coefficient in the IL with respect to the IT condition. Because the clustering coefficient summarizes a particular ROI node’s overall connection strength to the remaining regions, an increased clustering coefficient for these ‘heat nodes’ further corroborates the dominant role of higher-order processing in the WE deception task. The corresponding result for the NE task is shown in Table 6, significantly increased clustering coefficients were found for several higher-order execution areas, memory processing/comprehension areas and speech perception areas. This increase in higher-order execution areas does not violate the observed decreased intra-network connectivity in the corresponding network. Different from inter-group connectivity; the clustering coefficient for a node is a more general evaluation of connectivity with respect to the rest of the brain. The trend between them does not necessarily correlate. Among all nodes, the visual cortex (19L), perirhinal area (35L) and entorhinal cortex (34L) are common heat nodes with increased clustering coefficients for both tasks (WE vs. NE). This finding implies that deception involves memory processing unit with higher-order level of activities and this is likely to be a universal rule for various types of instructed lying.

### 4.3 Response time (correlation) analysis

Interestingly, the normalized event-related clustering coefficient for the inferior frontal gyrus (44R, Broca’s area) is positively correlated with the normalized response time in the WE-IL condition; this implies an increased amount of effort in executive control (as required in the IL condition), which leads to a delayed response time. Recent neuroimaging studies show BA44’s involvement in selective response suppression in a go/no-go task; BA44 is therefore believed to play an important role in the suppression of response tendency [76]. The normalized event-related clustering coefficients for precuneus (7L) and angular gyrus (39R), which are associated with comprehension/memory-processing in this study, show positive correlations with the normalized response time in the IT condition, indicating that comprehension/memory-processing could be processes that primarily contribute to the response time in this condition. However, a reverse trend of correlation was found for the NE task. The normalized event-related clustering coefficient of the secondary auditory cortex (42L) was found to be marginally negatively correlated with the response time in the NE-IL condition. Additionally, the normalized event-related clustering coefficients of the somatosensory association cortex (5L, 5R) were found to be negatively correlated with the response time in the NE-IT condition. It is known that both the secondary auditory cortex (42L) and the somatosensory association cortex (5L, 5R) are involved in sensory-level speech perception. For both regions, increased clustering coefficients in the IL condition imply their role in facilitating the semantic processing of stimuli. This largely explains the variation of response times between the NE-IL and the NE-IT conditions. The dominancy by speech perception in both conditions may be due to the subjects’ not having experiential knowledge of the stimuli, which requires them to spend even more time on speech perception. This also implies that well-designed probe stimuli (i.e., questions) play an important role in inducing neuronal features regarding the semantic processing during the lying task.

## Conclusion

The results of this study have provided intuitive evidence for distinguishable features of instructed lying with respect to instructed truth-telling conditions in network scale from EEG signals analysis in both the WE and the NE tasks during the stimulus delivery phase (SDP). The data analyzed with connectivity analysis/network analysis in the source space indicated contrastive network connection and nodal clustering coefficient patterns between the IL and IT conditions. Importantly, this study identified several fundamental processing units during the SDP that are common to all of the conditions. However, the involvement of these networks should be thoroughly investigated in the future with more instructed lying/truth-telling tasks and imaging modalities. As predicted, the major differences between the IL and IT conditions exist within the intra- and inter- group connectivity patterns. In addition, response time has been correlated with several key nodes’ clustering coefficients. As such, this study has provided a systematic level of understanding of the neural mechanisms underlying the IL and IT tasks based on a unique time window (SDP) with network analysis, and the results may be of great importance for future lie detection related researches and applications.

## Supporting Information

S1 Table Example questions for the instructed lying and truth-telling conditions in the WE task. (DOCX)

S2 Table Example questions for the instructed lying and truth-telling conditions in the NE task. (DOCX)
