# Supplementary material for: An Electroencephalography Network and Connectivity Analysis for Deception in Instructed Lying Tasks
Source: PLoS One. 2015 Feb 13;10(2):e0116522. doi: 10.1371/journal.pone.0116522 (PMC4332664; doi:10.1371/journal.pone.0116522)
Supplement: S1 Table — (DOCX) [file pone.0116522.s001.docx]

Table S1. Example questions for the instructed lying and truth-telling conditions in the WE task

| Instructed lying questions in the WE task | Instructed truth-telling questions in the WE task |
| --- | --- |
| Which year did you enter your university?  What is your major in your university?  What is your best friend’s nationality?  Which country’s food do you like most?  What kind of weather do you like most?  How do you travel to school every day? | How much is your living cost per month?  How many email accounts do you have?  What is your favorite brand of cellphone?  What drink is your favorite type of drink?  Which country are your parents living in?  When do you usually get up in the morning? |
