# Supplementary material for: An Electroencephalography Network and Connectivity Analysis for Deception in Instructed Lying Tasks
Source: PLoS One. 2015 Feb 13;10(2):e0116522. doi: 10.1371/journal.pone.0116522 (PMC4332664; doi:10.1371/journal.pone.0116522)
Supplement: S2 Table — (DOCX) [file pone.0116522.s002.docx]

Table S2. Example questions for the instructed lying and truth-telling conditions in the NE task

| Instructed lying questions in the NE task | Instructed truth-telling questions in the NE task |
| --- | --- |
| What is your position in your previous company?  When did you start to learn TANK software?  What is your salary in your previous company?  How long have you worked in this field?  What course have you learned for this area?  When did you go to U.S for an internship there? | Have you been to Germany for any training?  Have you received any certificate for this field?  Which software can you use for design?  Which countries have you been for business?  Which client are you most familiar with?  How many companies have you worked for? |
